# Chrysosplenol D can inhibit the growth of prostate cancer by inducing reactive oxygen species and autophagy

**DOI:** 10.1002/iid3.1061

**Published:** 2023-10-30

**Authors:** Haoyu Zhang, Zhixin You, Yilei Li, Cheng Gao, Yuhao Wang, Xiaoxiang Zhang

**Affiliations:** ^1^ Department of Urology The Second People's Hospital of Kunshan Kunshan Jiangsu China

**Keywords:** apoptosis, autophagy, chrysosplenol D (CHD), oxidative stress, prostate cancer

## Abstract

**Objective:**

To uncover the effects of chrysosplenol D (CHD) on the progression of prostate cancer in vitro as well as in mice.

**Methods:**

DU145 and PC‐3 cells were treated with increasing doses of CHD for 24, 48, or 72 h. Cell Counting Kit‐8 (CCK‐8) and colony formation assays were conducted to confirm the effects of CHD on cell viability. Flow cytometry (FCM) and immunostaining assays showed the effects of CHD on cell apoptosis and oxidative stress. Immunoblot was performed to detect the effects of CHD on autophagy. Besides, tumor growth assays were conducted to confirm the role of CHD in tumor growth in mice. GraphPad 6.0 was used for statistical analysis. All data were represented as mean ± SD. *p* < .05 and the significant difference was indicated by an asterisk.

**Results:**

CHD suppressed the viability of prostate cancer cells in CCK‐8 assays, decreased colony number in colony formation assays, and induced cell apoptosis in FCM and immunostaining assays. CHD also restrained the oxidative stress with a decreased 2′‐7′‐dichlorofluorescein diacetate staining intensity. CHD restrained the autophagy of prostate cancer cells, as well as suppressed tumor growth in mice.

**Conclusion:**

CHD could serve as a drug for prostate cancer.

## 1 INTRODUCTION

Prostate cancer is an extremely common type of malignancy in men.^1^ Its pathogenic factors have not been fully elucidated.^2^ Patients with prostate cancer have no obvious symptoms in the early stage, but they could have pain when urinating, hematuria, lumbar acid, pelvic pain, and other symptoms in the advanced stage.^3^ Currently, there are several treatments for prostate cancer at different stages. However, most treatments can cause serious side effects.^4^ The search for new treatments for prostate cancer is crucial.^5^, ^6^ In recent years, more and more natural active ingredients extracted from traditional Chinese medicinal materials have been used to combat cancer.^7^, ^8^ To combat prostate cancer, more effective targets and drugs are still needed. Inflammation is related to the onset of prostate cancer and recurrence after radical surgery. Therefore, controlling inflammation to a certain extent may be related to controlling the progression of prostate cancer and affecting the prognosis. However, drug therapy is still essential to improve patient outcomes and inflammatory responses.

Chrysosplenol D (CHD) is a flavonoid that can be isolated from traditional Chinese herbs such as *Artemisia annularis* and ground cover.^9^ CHD has anti‐inflammatory as well as anticancer properties.^10^, ^11^ For example, CHD protects mice from lipopolysaccharide‐induced acute lung injury by downregulating oxidative stress and inflammation, as well as apoptosis by inhibiting the TLR4‐MAPK/NF‐κB pathway.^11^ Experiments proved that CHD inhibited the proliferation of breast cancer cells and induced peroxide accumulation, mitochondrial membrane potential loss, and apoptosis.^12^ CHD induces apoptosis in TNBC cells.^12^ CHD induces cell cycle arrest with accumulation of cells at the S phase.^13^ CHD also exhibits antitrypanosomal activities.^14^, ^15^ CHD induces DNA damage in non‐small cell lung cancer by binding topo II‐α‐DNA and reducing topo II‐α activity.^13^ In addition, CHD inhibits the progression of oral squamous cell carcinoma by inducing autophagy and apoptosis.^13^, ^16^ The role of CHD in prostate cancer is unclear. Here, we investigated the role of CHD in the progression of prostate cancer and first found its effects on prostate cancer.

## 2 MATERIALS AND METHODS

### 2.1 Cell culture

Human DU145 (BFN60700106) and PC‐3 cells (BFN608007263; Bluefbio) were cultured in RPMI‐1640 medium supplemented with 10% fetal bovine serum (FBS) (both from Thermo Fisher Scientific Inc.) and 1% penicillin and streptomycin in a 5% CO_2_ incubator. Cells were treated with increasing doses of CHD (CAS No.: 14965‐20‐9, HY‐N6007, purchased from MCE, purity >98%) for 24, 48, or 72 h. Cells were also treated with *N*‐acetylcysteine (NAC; 50 mM, purchased from MCE) or 3‐methyladenine (3‐MA; 20 mM, purchased from MCE) for 24 h.

### 2.2 Cell viability

Cells were plated into 96‐well plates at a density of 1 × 10^3^ cells/well. After treatment with CHD, cell counting kit‐8 (CCK‐8) (Beyotime) was added to cells following rinsing with phosphate‐buffered saline (PBS). Cells were incubated for 4 h before the measurement of the OD450 value.

### 2.3 Colony formation assay

DU145 and PC‐3 cells were plated into the 6‐well plates (1000 cells/well) as well as maintained in a media (10% FBS) for 14 days at 37°C. Then, cells were fixed with paraformaldehyde for 20 min as well as stained with 0.1% crystal violet for 20 min. Afterward, the cells were photographed.

### 2.4 Cell apoptosis

Annexin V/propidium iodide (PI) apoptosis detection was conducted (Sigma‐Aldrich). Cells were digested and mixed in a buffer containing Annexin V as well as PI for 5 min. Cell proportions were analyzed using a FACSCalibur flow cytometer and CellQuest Pro 5.1 (BD Biosciences Inc.).

### 2.5 Immunoblot assay

Cells were lysed in a buffer containing 1% Triton X‐100, 150 mM NaCl, and 50 mM Tris (pH 7.5). Proteins were then transferred onto polyvinylidene difluoride membranes (Millipore Sigma), which were blocked at room temperature for 2 h in Tris‐buffered saline containing 0.2% Tween‐20 and 5% nonfat milk. The membranes were then incubated with primary antibodies of LC3 (ab192890, 1:500; Abcam), NQO1 (ab80588, 1:1000; Abcam), AKT (ab8805, 1:1000; Abcam), p‐AKT (ab38449, 1:500; Abcam), mTOR (ab134903, 1:500; Abcam), p‐mTOR (ab109268, 1:500; Abcam), and β‐actin (ab8226, 1:3000; Abcam) for 1 h. Subsequently, the membranes were incubated with secondary antibodies for 1 h. The blots were analyzed with an ECL kit.

### 2.6 Reactive oxygen species (ROS) assay

The cellular ROS level was detected using 2′‐7′‐dichlorofluorescein diacetate (Sigma‐Aldrich). Then, the cells were washed. Subsequently, slides were stained with 4′,6‐diamidino‐2‐phenylindole (1:3000; Sigma‐Aldrich) for 3 min. Coverslips were mounted with 90% glycerol in PBS and then examined. Cells were measured using ImageJ 9.0 software.

### 2.7 Enzyme‐linked immunosorbent assay (ELISA) assay

The concentrations of caspase‐3 in the cell lysates were measured with an ELISA kit (Beyotime) following the protocols.

### 2.8 Tumor growth in vivo assay

All animal procedures were approved by the Institutional Animal Care and Use Committee of the Second People's Hospital of Kunshan (Approval no. 2022‐13). A total of eight female nude mice (age, 8 weeks; weight, 22–24 g; *n* = 6 mice/group) were purchased from Beijing Vital River Laboratory Animal Technology Co., Ltd. The tumor growth in vivo assays were approved by the Institutional Animal Use Committee of the Second People's Hospital of Kunshan. DU145 (10^5^) cells were injected into nude mice. After 35 days, all of the mice were killed by cervical dislocation, and the lack of heartbeat was confirmed to validate death. The tumors were analyzed between control (PBS) and CHD (20 mg/kg/day) treatment (for 7 days) groups. Body weight and tumor weight were measured. The messenger RNA levels and weight were calculated according to the average value of the six nude mice in each group. The formula for calculating the subcutaneous tumor volume in mice is *V* = (*L* × *W* ^2^)/2, where *V* represents the tumor volume, *L* represents the tumor length, and *W* represents the tumor width.

### 2.9 Statistical analysis

Data were analyzed using GraphPad 6.0 software (GraphPad Software Inc., National Institutes of Health) and expressed as mean ± SD. The unpaired Student's *t*‐test was used to determine the statistical significance between the two groups. One‐way analysis of variance followed by Tukey's post hoc test was used for multiple comparisons. *p* < .05 was considered statistically significant.

## 3 RESULTS

### 3.1 CHD inhibited the proliferation of prostate cancer cells

**Figure 1:**
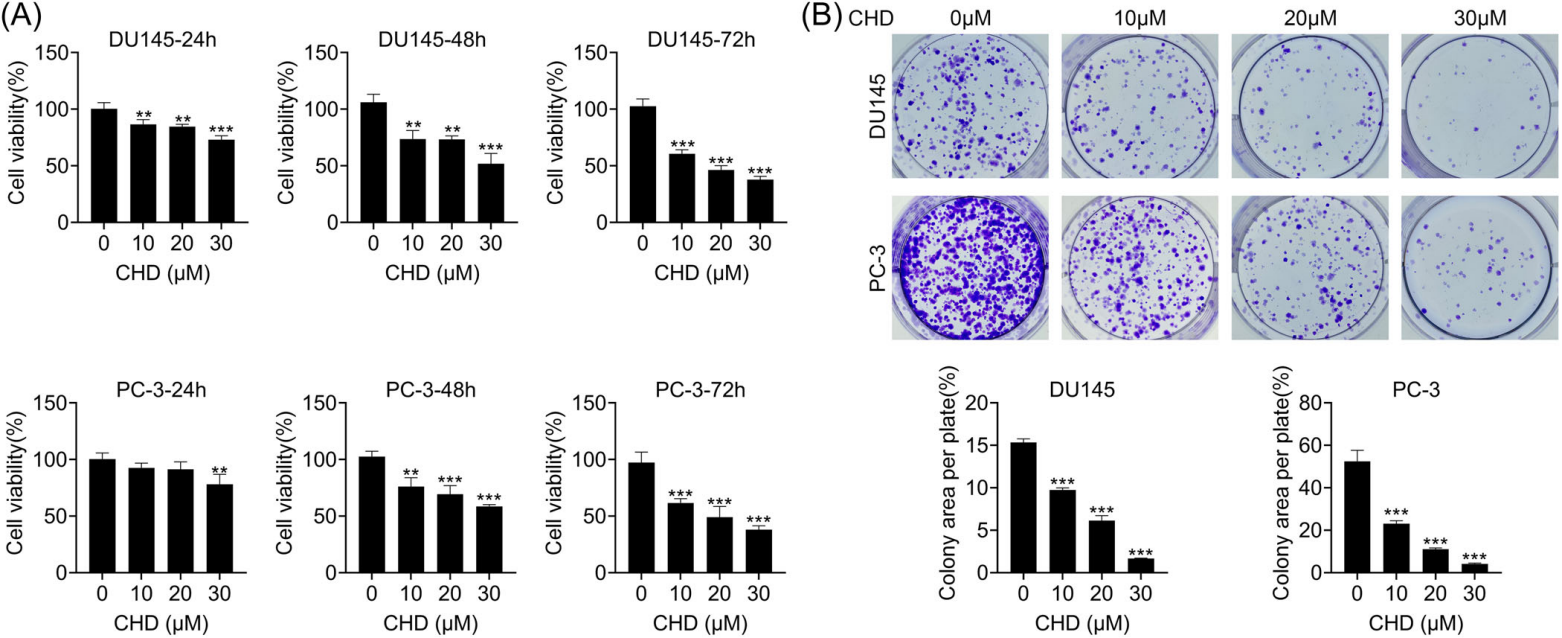
Chrysosplenol D (CHD) inhibited the proliferation of prostate cancer cells. (A) Cell Counting Kit‐8 assays showed the proliferation capacity of DU145 and PC‐3 cells treated with CHD for 24, 48, and 72 h, and the OD450 value was measured. (B) Colony formation assays showed the colony numbers in DU145 and PC‐3 cells treated with CHD at concentrations of 0, 10, 20, and 30 μM, and colony numbers were counted. Data are presented as mean ± SD. **p < .01; ***p < .001.

The prostate cancer cell lines, including DU145 and PC‐3, were treated with CHD at concentrations of 10, 20, and 30 μM. CCK‐8 assays showed the proliferation capacity of DU145 and PC‐3 cells treated with CHD for 24, 48, and 72 h (Figure 1A), with a decreased OD450 value (Figure 1A). Also, colony formation assays showed decreased colony numbers in DU145 and PC‐3 cells treated with CHD at concentrations of 10, 20, and 30 μM (Figure 1B). We thought CHD inhibited the proliferation of prostate cancer cells.

### 3.2 CHD induced apoptosis in prostate cancer cells

**Figure 2:**
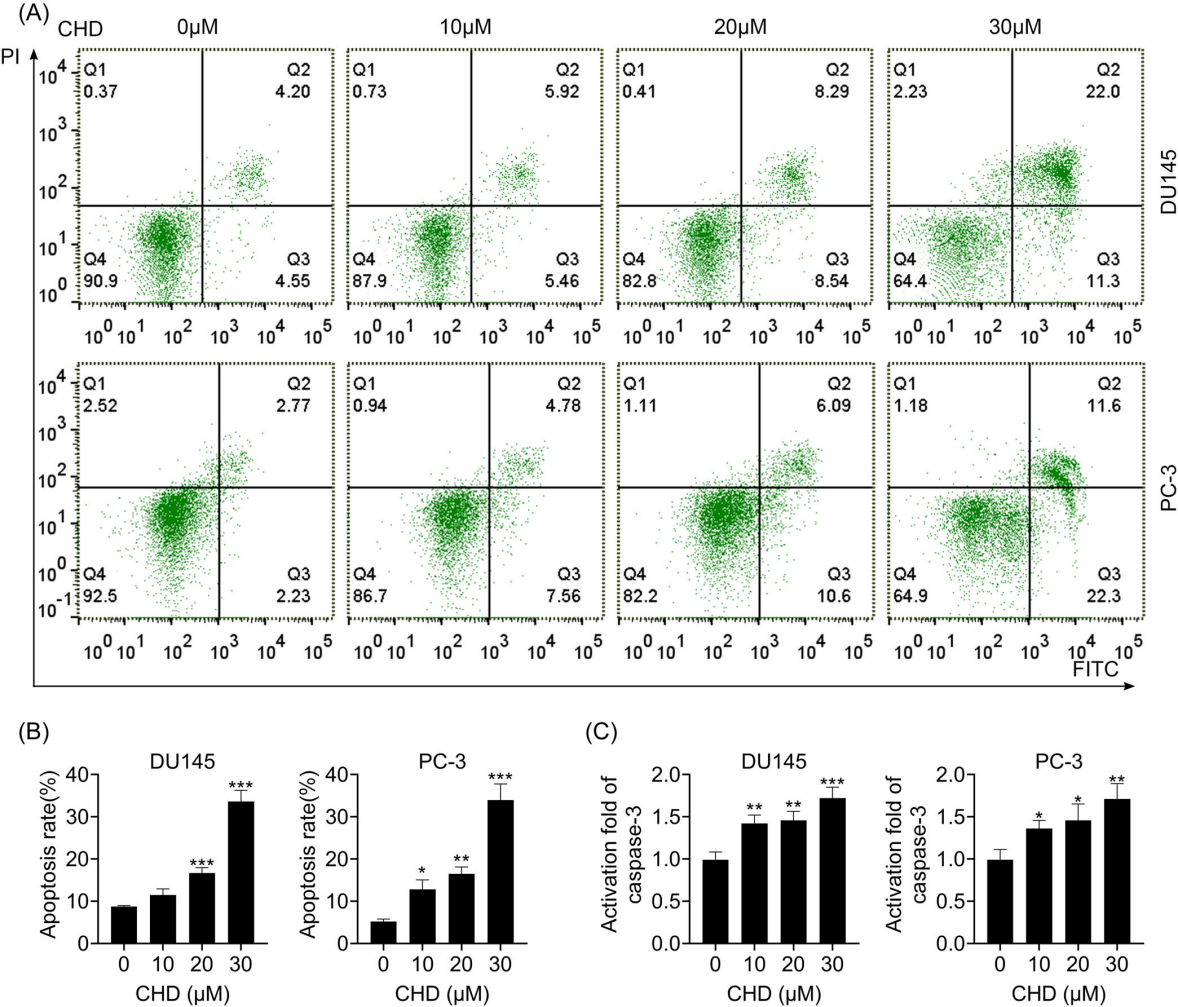
Chrysosplenol D (CHD) induced apoptosis in prostate cancer cells. (A) Flow cytometry assays were performed to investigate the apoptosis of DU145 and PC‐3 cells treated with CHD at concentrations of 0, 10, 20, and 30 μM. (B) The apoptosis rates of cells were quantified. (C) Caspase‐3 activity of DU145 and PC‐3 cells treated with CHD at concentrations of 0, 10, 20, and 30 μM was detected through enzyme‐linked immunosorbent assay assays. Data are presented as mean ± SD. *p < .05; **p < .01; ***p < .001.

The effect of CHD on cell apoptosis was measured by flow cytometry. Flow cytometry (FCM) assays were performed to investigate the apoptosis of DU145 and PC‐3 cells treated with CHD at concentrations of 10, 20, and 30 μM (Figure 2A). CHD treatment suppressed the apoptosis of DU145 and PC‐3 cells at concentrations of 10, 20, and 30 μM, with increased apoptosis rates (Figure 2B). In addition, the activity of caspase‐3 was evaluated. An increased activity of caspase‐3 was noticed in DU145 and PC‐3 cells when stimulated by CHD (Figure 2C). These data verified that CHD induced apoptosis in prostate cancer cells.

### 3.3 CHD restrained the oxidative stress of prostate cancer cells

**Figure 3:**
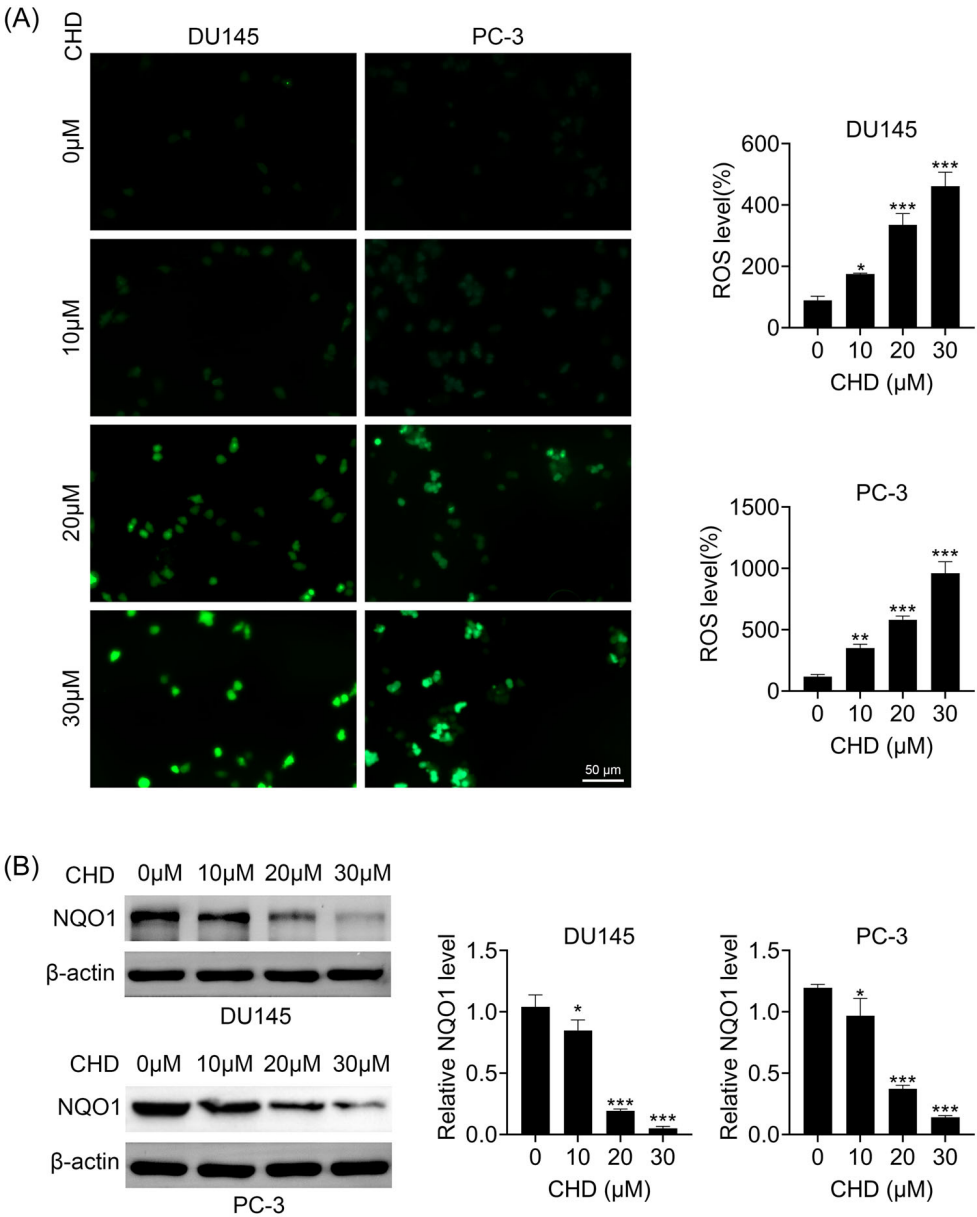
Chrysosplenol D (CHD) restrained the oxidative stress of prostate cancer cells. (A) Immunostaining assays showed the effects of CHD on the oxidative stress of DU145 and PC‐3 cells treated with CHD at concentrations of 0, 10, 20, and 30 μM. The reactive oxygen species (ROS) levels were measured. (B) Immunoblot assays showed the effects of CHD on the expression of NQO1 in DU145 and PC‐3 cells treated with CHD at concentrations of 0, 10, 20, and 30 μM. Data are presented as mean ± SD. *p < .05; **p < .01; ***p < .001.

We then investigated whether CHD affected the oxidative stress of prostate cancer cells through immunostaining and immunoblot assays. Interestingly, CHD treatment increased the ROS levels of DU145 and PC‐3 cells (Figure 3A). Immunoblot assays showed that CHD treatment decreased the expression of NQO1 in DU145 and PC‐3 cells (Figure 3B). We further found that the inhibitor of oxidative stress, NAC, could prevent the inhibition of proliferation and the promotion of apoptosis caused by CHD in prostate cancer cells, confirmed by immunostaining, CCK‐8, FCM, and immunoblot assays (Supporting Information: Figure S1A–D). Therefore, we thought CHD restrained the oxidative stress of prostate cancer cells.

### 3.4 CHD suppressed autophagy in prostate cancer cells

**Figure 4:**
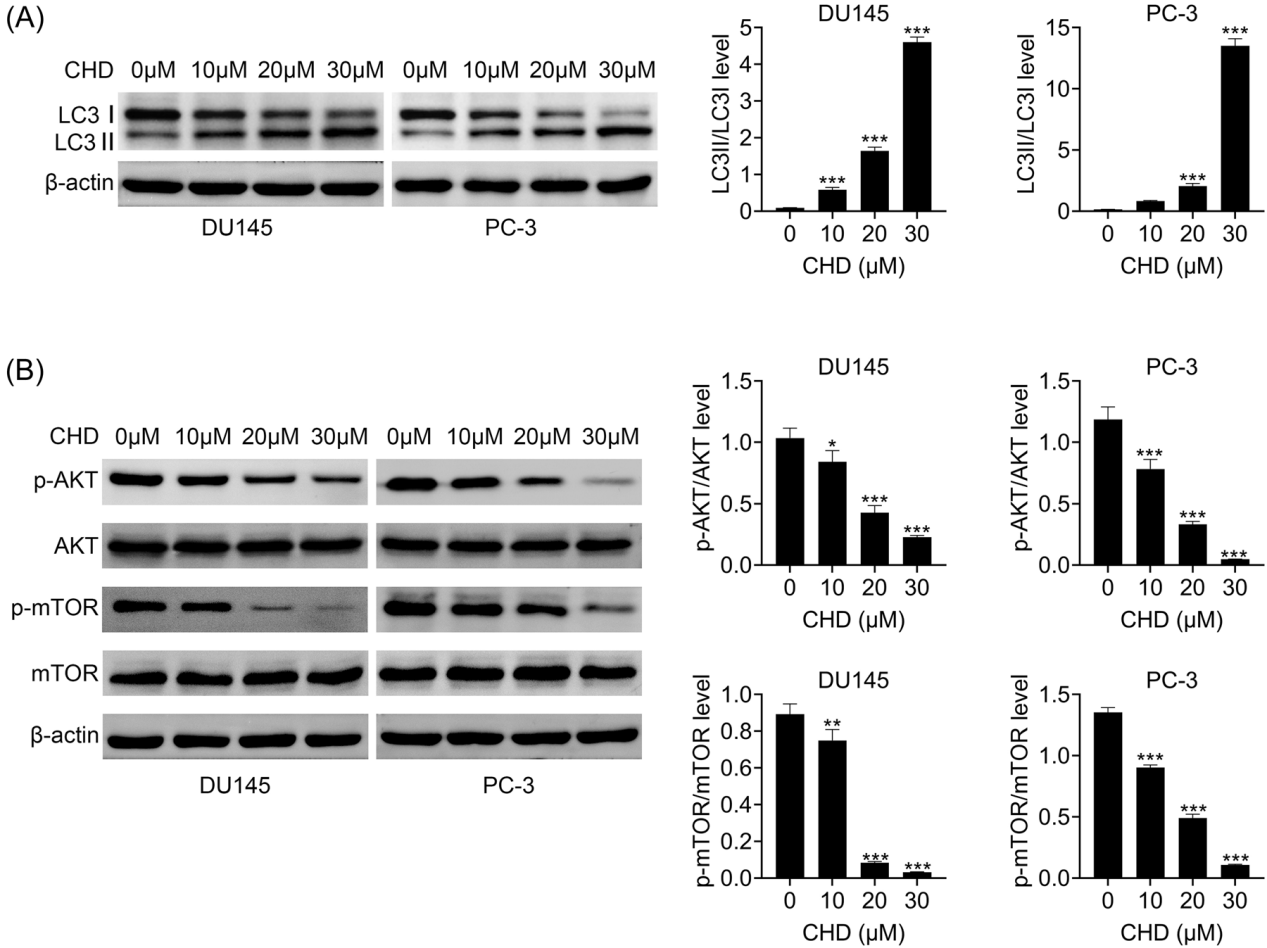
Chrysosplenol D (CHD) suppressed the autophagy of prostate cancer cells. (A) Immunoblot assays showed the effects of CHD on the expression of LC3 in DU145 and PC‐3 cells treated with CHD at concentrations of 0, 10, 20, and 30 μM. (B) Immunoblot assays showed the effects of CHD on the expression and phosphorylation of AKT and mTOR in DU145 and PC‐3 cells treated with CHD at concentrations of 0, 10, 20, and 30 μM. Data are presented as mean ± SD. *p < .05; **p < .01; ***p < .001.

Through immunoblot assays we noticed that CHD treatment decreased the LC3‐I expression and increased the LC3‐II expression in DU145 and PC‐3 cells, suggesting a decrease in the LC3‐II/LC3‐I level and inhibition of autophagy (Figure 4A). We further found that the inhibitor of autophagy, 3‐MA, could prevent the inhibition of proliferation and the promotion of apoptosis caused by CHD in prostate cancer cells, confirmed by CCK‐8 and FCM assays (Figure 4E,F). The mTOR/AKT pathway is a key regulator of cell autophagy. We detected the effects of CHD on this pathway through immunoblot assays. We noticed that CHD treatment decreased the phosphorylation levels of AKT and mTOR in DU145 and PC‐3 cells, suggesting the inhibition of autophagy (Figure 4B). Therefore, CHD suppressed autophagy in prostate cancer cells.

### 3.5 CHD inhibited tumor growth of prostate cancer in mice

**Figure 5:**
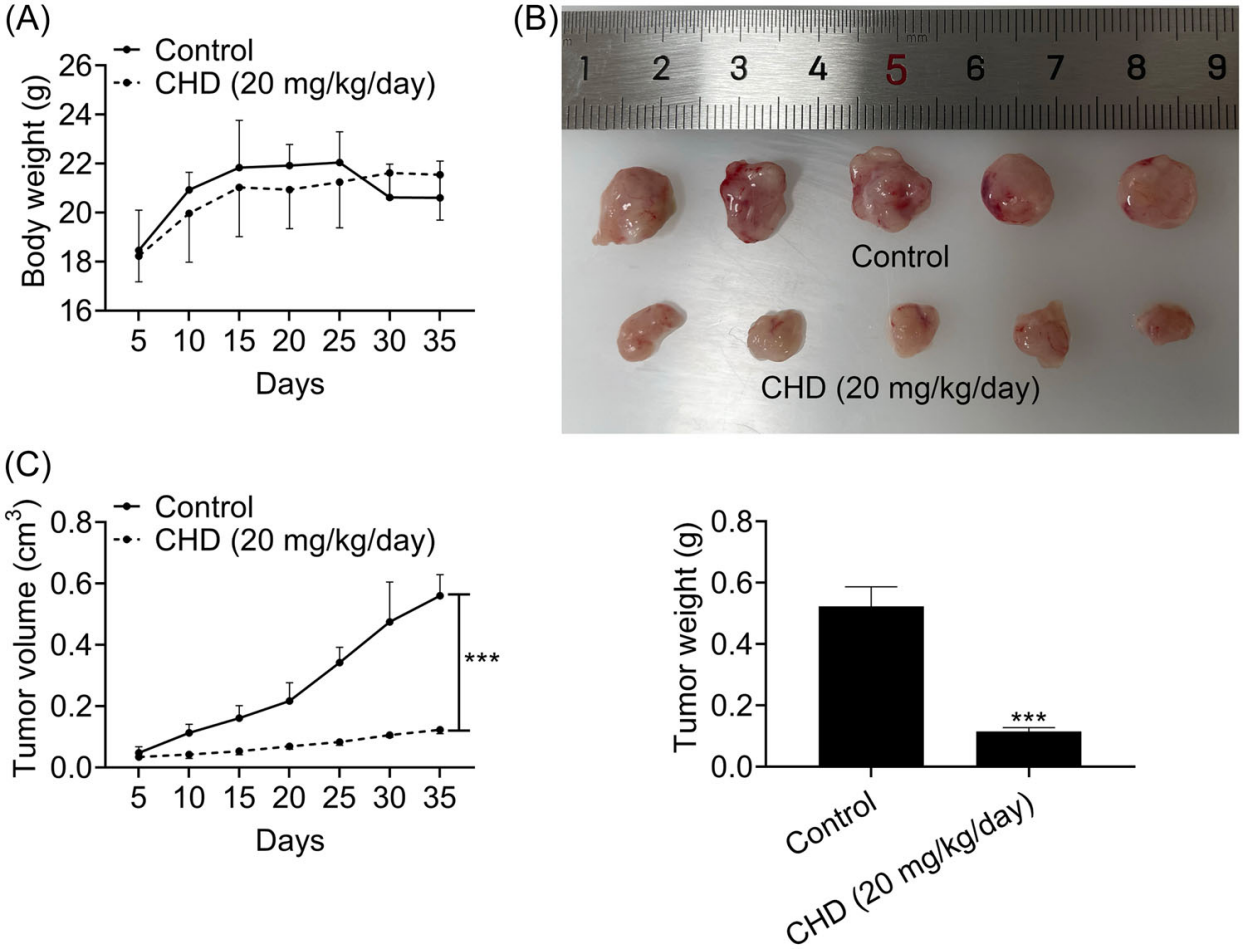
Chrysosplenol D (CHD) inhibited tumor growth of prostate cancer in mice. DU145 cells were injected subcutaneously into the abdomen of nude mice (*n* = 6 for each group) upon the indicated treatment to induce tumor growth. Body weight was shown in panel A, tumor images were shown in panel A, and tumor volume and weight were shown in panels C and D, respectively. Data are presented as mean ± SD. ***p < .001.

To detect whether CHD inhibits the growth of prostate cancer in mice, CHD treatment was given at a concentration of 20 mg/kg/day. The body weight and tumor volume were assessed every 5 days for 35 days. We noticed that CHD treatment has modest effects on body weight (Figure 5A). Interestingly, CHD treatment dramatically restrained prostate cancer growth, with a decreased tumor volume and weight (Figure 5B–D). Therefore, CHD inhibited the growth of prostate cancer in mice.

## 4 DISCUSSION

The true genetic and molecular pathophysiology of prostate cancer is a complex topic.^17^ Unlike breast cancer, there is no highly permeable and dominant genetic mutation that accounts for the majority of prostate cancer cases.^2^ Therefore, there is huge room for future therapeutic innovation, and customized genetic analysis is likely to play a role in reducing prostate cancer prevalence.^18^ Dysregulation of apoptosis is important in the pathogenesis of prostate cancer and is thought to be a key driver of the exponential growth of tumor cells.^19^ Expanding our understanding of the pathogenesis of prostate cancer is critical. Looking for truly predictive markers and tissue assessments can benefit to understanding the potential clinical outcomes of prostate tumors and developing highly effective treatments with modest adverse effects caused by unnecessary procedures and diagnoses.^20^ Drug treatment for prostate cancer, including endocrine therapy and chemotherapy drugs, has limited effect on patients with advanced tumors.^18^ Here, we noticed that a new drug, CHD, could suppress prostate cancer progression in vitro as well as in mice. We therefore thought it could serve as a drug for prostate cancer treatment.

Notably, inflammation is correlated to the occurrence of prostate cancer as well as the recurrence after radical prostate cancer. Therefore, mediating inflammation may be closely related to the progression of prostate cancer, affecting the ultimate surgical outcome. Our data confirmed that CHD inhibited the growth of prostate cancer by inducing ROS and autophagy. We should further confirm whether CHD could affect the inflammation of prostate cancer cells in vitro as well as in mice.

CHD protects mice from LPS‐induced acute lung injury by downregulating oxidative stress, and inflammation, as well as apoptosis by inhibiting the TLR4‐MAPK/NF‐κB pathway.^11^ In vitro and in vivo experiments proved that CHD inhibited the proliferation of breast cancer cells and induced peroxide accumulation, mitochondrial membrane potential loss, and apoptosis.^12^ In addition, CHD induced apoptosis by suppressing topoisomerase IIα in human lung cancer cells.^13^ All of these studies confirmed the antitumor effects of CHD. However, the precise mechanism needs further study.

Autophagy is a process in which eukaryotic cells recycle their own biomacromolecules and organelles through lysosomes.^21^ In the process of tumor pathogenesis, autophagy plays a “double‐sided role,” which can not only fight cancer but also promote cancer development.^22^ To explore the regulatory mechanism of autophagy in prostate cancer and develop related drugs for autophagy may provide a new method for the treatment of prostate cancer.^23^ We also revealed that CHD affected the progression of prostate cancer by suppressing autophagy. In addition, oxidative stress is related to the occurrence of prostate hyperplasia and prostate cancer.^24^ Studies have shown that there are differences in the expression of oxidative stress indicators in BPH and prostate cancer cells, and the oxidative stress response in prostate cancer cells is stronger than that in BPH.^24^ Therefore, we detected the effects of CHD in the oxidative stress of prostate cancer cells. As was expected, we found CHD suppressed the oxidative stress of prostate cancer cells. Inflammation plays an important role in the initiation and deterioration of PCa, while autophagy can act as a promoter or inhibitor of prostate cancer, and androgen can promote the growth of prostate cancer cells through androgen receptors. Autophagy also plays an important role in the treatment of prostate cancer. Therefore, both inflammation and autophagy play an important role in prostate cancer, and CHD inhibits prostate cancer progression mainly by affecting inflammation and autophagy.^23^

The limitation of this study is the lack of in‐depth mechanism research, and the next step is to systematically investigate the signaling pathway through which CHD affects prostate cancer. Relevant downstream proteins and pathways can be analyzed in combination with proteomics and transcriptomics and validated at the cellular and animal levels. We will perform the in vivo assays in the future to confirm the effects of CHD on tumor growth in mice. We will further perform the immunoblot assays to confirm the mechanism underlying CHD suppressing prostate cancer progression.

In summary, we revealed that CHD inhibited the proliferation of prostate cancer cells, induced cell apoptosis, ROS production, and autophagy levels. We thought all of them suppressed the progression of prostate cancer. In vivo experiments further demonstrated that CHD inhibited the proliferation of prostate cancer.

## AUTHOR CONTRIBUTIONS

All authors contributed to the study conception and design. Material preparation and the experiments were performed by Haoyu Zhang. He was involved in conceptualization; investigation, and methodology. Data collection and analysis were performed by Zhixin You, Yilei Li, Cheng Gao, and Yuhao Wang. The first draft of the manuscript was written by Xiaoxiang Zhang and all authors commented on previous versions of the manuscript. All authors read and approved the final manuscript.

## CONFLICT OF INTEREST STATEMENT

The authors declare no conflict of interest.

## ETHICS STATEMENT

Ethical approval was obtained from the Ethics Committee of the Second People's Hospital of Kunshan (Approval no. 2022‐13).

## Supporting information

Supporting information.

## Data Availability

All data generated or analyzed during this study are included in this published article. The data sets used and/or analyzed during the present study are available from the corresponding author upon reasonable request.

## References

[1] Kim TH , Park JM , Kim MY , Ahn YH . The role of CREB3L4 in the proliferation of prostate cancer cells. Sci Rep. 2017;7:45300. 28338058 10.1038/srep45300 PMC5364418

[2] Shen P , Sun J , Xu G , et al. KLF9, a transcription factor induced in flutamide‐caused cell apoptosis, inhibits AKT activation and suppresses tumor growth of prostate cancer cells. Prostate. 2014;74(9):946‐958. 24737412 10.1002/pros.22812

[3] Cui F , Hu J , Xu Z , Tan J , Tang H . Overexpression of NCAPH is upregulated and predicts a poor prognosis in prostate cancer. Oncol Lett. 2019;17(6):5768‐5776. 31186803 10.3892/ol.2019.10260 PMC6507296

[4] Tang Z , Pilié PG , Geng C , et al. ATR inhibition induces CDK1‐SPOP signaling and enhances anti‐PD‐L1 cytotoxicity in prostate cancer. Clin Cancer Res. 2021;27(17):4898‐4909. 34168048 10.1158/1078-0432.CCR-21-1010 PMC8456453

[5] Kim JK , Kim JY , Kim HJ , et al. Scoparone exerts anti‐tumor activity against DU145 prostate cancer cells via inhibition of STAT3 activity. PLoS One. 2013;8(11):e80391. 24260381 10.1371/journal.pone.0080391 PMC3829856

[6] Camilloni A , Nati G , Maggiolini P , Romanelli A , Latina R . Chronic non‐cancer pain in primary care: an Italian cross‐sectional study. Signa Vitae. 2021;7(2):54‐62.

[7] Lei L , Yu L , Fan W , Hao X . Prostate cancer small extracellular vesicles participate in androgen‐independent transformation of prostate cancer by transferring let‐7a‐5p. Heliyon. 2022;8(12):e12114. 36578414 10.1016/j.heliyon.2022.e12114 PMC9791359

[8] Luan Y , Guo G , Luan Y , Yang Y , Yuan R . Single‐cell transcriptional profiling of hearts during cardiac hypertrophy reveals the role of MAMs in cardiomyocyte subtype switching. Sci Rep. 2023;13(1):8339. 37221368 10.1038/s41598-023-35464-2 PMC10205799

[9] Kraus G , Roy S . Direct synthesis of chrysosplenol D. J Nat Prod. 2008;71(11):1961‐1962. 18855445 10.1021/np800423j

[10] Behl T , Kaur I , Kotwani A . Implication of oxidative stress in progression of diabetic retinopathy. Surv Ophthalmol. 2016;61:187‐196. 26074354 10.1016/j.survophthal.2015.06.001

[11] Zhang Q , Feng A , Zeng M , et al. Chrysosplenol D protects mice against LPS‐induced acute lung injury by inhibiting oxidative stress, inflammation, and apoptosis via TLR4‐MAPKs/NF‐κB signaling pathways. Innate Immun. 2021;27(7‐8):514‐524. 34806444 10.1177/17534259211051069 PMC8762090

[12] Lang SJ , Schmiech M , Hafner S , et al. Chrysosplenol d, a flavonol from *Artemisia annua*, induces ERK1/2‐mediated apoptosis in triple negative human breast cancer cells. Int J Mol Sci. 2020;21(11):4090. 32521698 10.3390/ijms21114090 PMC7312517

[13] Fu C , Zhang K , Wang M , Qiu F . Casticin and chrysosplenol D from *Artemisia annua* L. induce apoptosis by inhibiting topoisomerase IIα in human non‐small‐cell lung cancer cells. Phytomedicine. 2022;100:154095. 35398735 10.1016/j.phymed.2022.154095

[14] Li YJ , Guo Y , Yang Q , et al. Flavonoids casticin and chrysosplenol D from *Artemisia annua* L. inhibit inflammation in vitro and in vivo. Toxicol Appl Pharmacol. 2015;286(3):151‐158. 25891417 10.1016/j.taap.2015.04.005

[15] Ng CL , Lee SE , Lee JK , et al. Solubilization and formulation of chrysosplenol C in solid dispersion with hydrophilic carriers. Int J Pharm. 2016;512(1):314‐321. 27593897 10.1016/j.ijpharm.2016.08.062

[16] Hsieh MJ , Lin CC , Lo YS , Chuang YC , Ho HY , Chen MK . Chrysosplenol D triggers apoptosis through heme oxygenase‐1 and mitogen‐activated protein kinase signaling in oral squamous cell carcinoma. Cancers. 2021;13(17):4327. 34503136 10.3390/cancers13174327 PMC8430639

[17] Shahid M , Kim M , Lee MY , et al. Downregulation of CENPF remodels prostate cancer cells and alters cellular metabolism. Proteomics. 2019;19(11):e1900038. 30957416 10.1002/pmic.201900038 PMC6633900

[18] Zambrano IA , Hwang S , Basak R , et al. Patterns of multispecialty care for low‐ and intermediate‐risk prostate cancer in the use of active surveillance. Urol Oncol. 2023;41:388.e1‐388.e8. 10.1016/j.urolonc.2023.04.024 37286404

[19] Pieramico S , Castro R , Aguiar S , et al. A systematic review on the efficacy of CBT interventions for the mental and sexual health of survivors of prostate cancer. Sex Med Rev. 2023. 10.1093/sxmrev/qead024. 37286525

[20] Patient‐Reported outcomes after monitoring, surgery, or radiotherapy for prostate cancer. N Engl J Med. 2023;388(23):2208. 37285545 10.1056/NEJMx230003

[21] Roperto S . Role of BAG3 in bovine deltapapillomavirus‐mediated autophagy. JCB. 2022;123(1):59‐64. 34889472 10.1002/jcb.30193

[22] Kögel D , Linder B , Brunschweiger A , Chines S , Behl C . At the crossroads of apoptosis and autophagy: multiple roles of the co‐chaperone BAG3 in stress and therapy resistance of cancer. Cells. 2020;9(3):574. 32121220 10.3390/cells9030574 PMC7140512

[23] Feng Y , Cao H , Song Z , Chen L , Wang D , Gao R . Qi Ling decoction enhances abiraterone treatment via suppression of autophagy in castration resistant prostate cancer. Aging. 2022;14(24):9942‐9950. 36541904 10.18632/aging.204427 PMC9831723

[24] Wu Z , Su M , Chen H , et al. Sinularin exerts anti‐cancer effects by inducing oxidative stress‐mediated ferroptosis, apoptosis, and autophagy in prostate cancer cells. Anti Cancer Agents Med Chem. 2023;23:1457‐1468. 10.2174/1871520623666230331083744 37005535

