# Supplementary figures and images for: Chrysosplenol D can inhibit the growth of prostate cancer by inducing reactive oxygen species and autophagy
Source: Immun Inflamm Dis. 2023 Oct 30;11(10):e1061. doi: 10.1002/iid3.1061 (PMC10614118; doi:10.1002/iid3.1061)

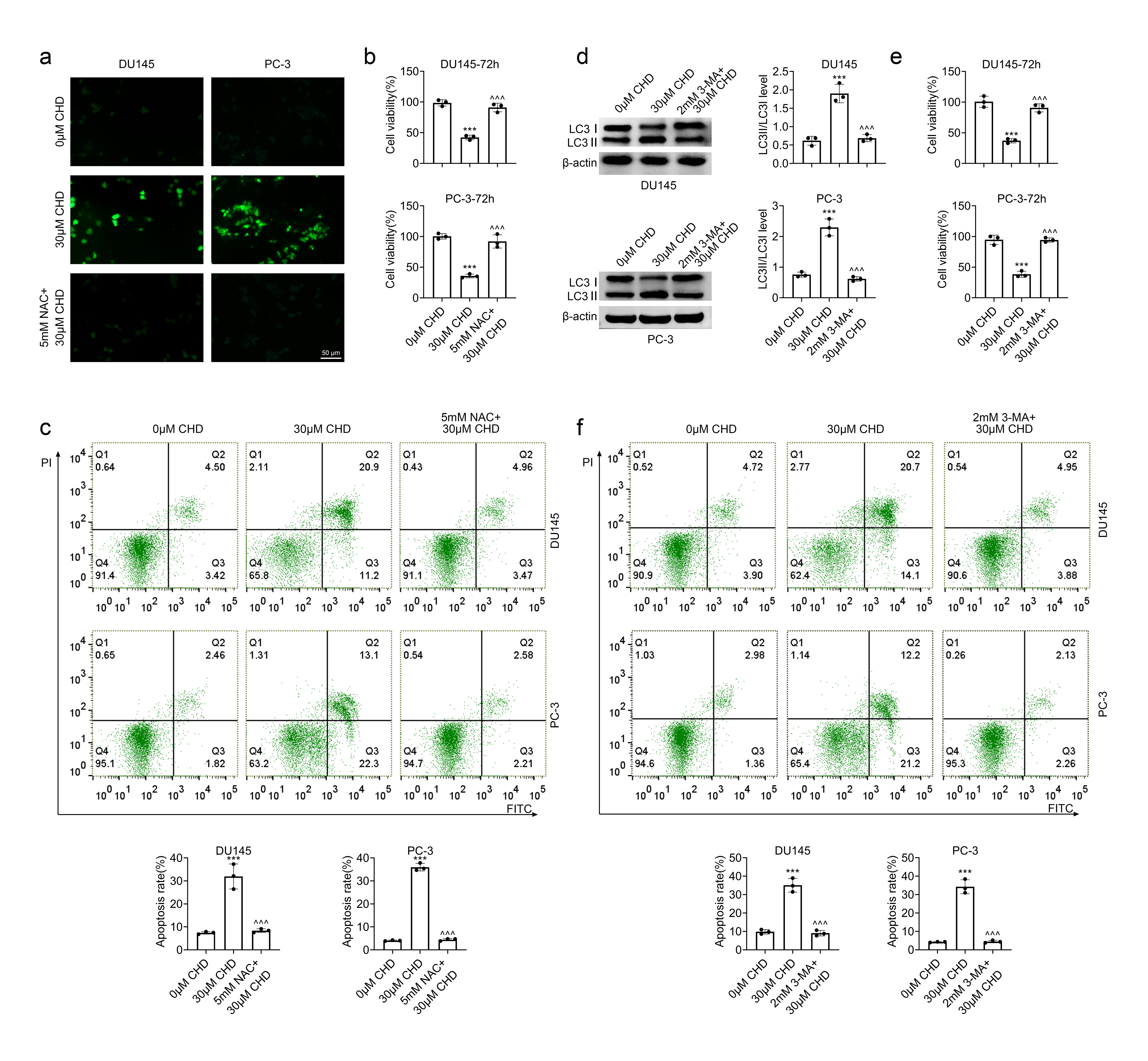

Supplement: Supplementary file 1 — Supporting information. [file IID3-11-e1061-s001.jpg]
